# Epidemiology of major incidents: an EMS study from Pakistan

**DOI:** 10.1186/1865-1380-4-48

**Published:** 2011-07-28

**Authors:** Hunniya Waseem, Luca Carenzo, Junaid Razzak, Rizwan Naseer

**Affiliations:** 1 Public Health Solutions Pakistan, Lahore, Pakistan; 2 Research Center in Disaster and Emergency Medicine, Università Del Piemonte Orientale "A. Avogadro", Novara, Italy; 3 Department of Emergency Medicine, Aga Khan University Hospital, Karachi, Pakistan; 4 Rescue 1122, Punjab Emergency Service, Lahore, Pakistan

## Abstract

**Background:**

A major incident is defined as an event that owing to the number of casualties has the potential to overwhelm the available resources. This paper attempts to describe the incidence and epidemiology of major incidents dealt with by a government-run emergency medical service (EMS) in the Punjab province of Pakistan, a developing country in South Asia. A major incident in this EMS is defined as any incident that produces three or more patients, or any incident in which extraordinary resources are needed.

**Methods:**

All the calls received by an EMS Rescue 1122 were studied over a 6-month period. Calls that were defined as major incidents were identified, and further details were sought from the districts regarding these incidents. Questions specifically asked were the type of incident, time of the incident, response time for the incident, the resources needed, and the number of dead and injured casualties. Retrospective data were collected from the submitted written reports.

**Results:**

Road traffic crashes (RTCs) emerged as the leading cause of a major incident in the province of Punjab and also led to the greatest number of casualties, followed by fire incidents. The total number of casualties was 3,380, out of which 73.7% were RTC victims. There was a high rate of death on the scene (10.4%). Certain other causes of major incidents also emerged, including violence, gas explosions and drowning.

**Conclusion:**

Road traffic crashes are the most common cause of a major incident in developing countries such as Pakistan. Injury prevention initiatives need to focus on RTCs.

## Summary Box

### What is already known ?

Major incidents overwhelm local emergency medical service. For planning purposes it is imperative to study the epidemiology and incidence of such incidents.

### What this paper adds?

Major incidents have not been studied in developing countries. We present findings of a study conducted by a trained pre-hospital service in a province of Pakistan. Road traffic crashes emerged as the most prevalent cause of a major incident in our study, and they cause the greatest number of casualties. The findings of this study will have implications for public health professionals who are dealing with injury prevention in the developing world.

## Introduction

Major incidents have been defined as "events that owing to the number and severity of live casualties require special arrangements" [1]. This definition encompasses a wide array of incidents that may include mass gatherings, train crashes, large road traffic crashes [RTCs] or bombings. Any incident that is unpredictable in nature, sudden and results in a significant number of injured who overwhelm the capacity of the emergency medical services is defined as a major incident [2]. The definition of a major incident thus varies across places and is dependent on the amount of resources available to the presenting casualties. Therefore, not only is the number of casualties important, but also the severity of injuries, the local resources and their capability of handling such an incident [3].

Pakistan is a developing country in South Asia with a struggling health-care infrastructure [4]. Like many other third world countries, emergency medical services both pre-hospital and hospital have long been neglected in Pakistan [5,6]. Traditionally, patients have been brought to the emergency departments by relatives or bystanders, or at most in rudimentary patient transport vehicles [7-9]. Even in major incidents, no on-site help or support has been available, and patients are often haphazardly collected and brought to the nearest Emergency Department [10,11].

In these circumstances it is perhaps not surprising that a definition of major incidents has never been decided upon, and no previous data exist about major incidents in this country.

This study was undertaken to describe the incidence and epidemiology of major incidents in Punjab over a 6-month time period.

## Methodology

### Study design

This was a retrospective review of all cases identified as "major incidents" by the Rescue 1122 service, a pre-hospital service in Punjab. A major incident in this service is defined as any incident that produces three or more patients, or an incident in which extraordinary resources are needed [12]. Rescue 1122 has defined this as a major incident because three or more victims mean that they have to mobilize extra resources and need more than one ambulance on the scene. They get called in for road traffic crashes[RTCs] as well as fire incidents and water rescues.

### Setting

Rescue 1122 is a pre-hospital care service functioning in the province of Punjab, Pakistan. This service initially started providing pre-hospital treatment and transfer to patients in fully equipped ambulances with trained emergency medical technicians [13]. With time, however, it expanded to include fire services, disaster teams and water rescue services [14]. It has started to play a role in appropriate triaging, and transferring of patients to hospitals after major incidents and disasters [15].

The public can activate the emergency medical service by dialing a toll-free number. Trained operators at the dispatch center receive the incident information from the public and dispatch the first ambulance, and may then call for more resources after an on-scene needs assessment has been performed. In case of a fire call, an ambulance always accompanies the fire engine.

### Data collection and analysis

Data were collected directly from Rescue 1122 Headquarters. Every time the service deals with a major incident in any district, an initial report reaches the Headquarters. After that, within 24 h the district faxes a complete report to the HQ comprising the time of the call, response time, nature of the emergency, number of casualties, their disposition and the resources used. The number of personnel involved as well as the name of the incident commander is also part of the report [12].

For the purpose of this study we used these reports. We especially concentrated on the nature of emergencies, the total number of dead and injured casualties, and their disposition. Data were analyzed using GraphPad Prism version 5.0 (GraphPad Software, San Diego, CA), tested for normality by Kolmogorov-Smirnov test, and expressed as mean and standard deviation or median and interquartile range according to the normal distribution or not. The comparison of numerical variables was performed using the Mann-Whitney test for nonparametric data. Values of *p *< 0.05 were considered significant.

## Results

During the 6-month study period, the Punjab 1122 Emergency Medical System responded to a total of 438 major incidents; 48.6% of these were related to road traffic crashes (RTCs), 26.7% to fires, 6.5% to building collapses, 7.5% to episodes of violence (including firearms injuries), 3.0% to drownings, 1.8% to high height falls, 1.4% to poisonings, 1.4% to explosive cylinder blasts, 1.2% to gas leaks, 1.2% to bomb explosions, 0.5% to train accidents and 0.2% to electric shocks. The total number of casualties resulting from these calls was 3,380. The majority of injuries were among the victims of RTCs (73.7%), followed by violence (7.7%), fires (5.3%), building collapse (3.5%) and bomb explosions (3.4%), and the remaining 6.4% were related to the other causes. Three hundred fifty-one patients (10.4%) were found dead on the scene, while 828 (24.5%) were discharged by the EMTs after first aid from the scene; 2,201 (65.1%) were transported to the nearest health facility, which in most cases was the district hospital. Of the 351 deaths, 46.2% were caused by road traffic crashes, 3.4% by fires, 31.6% by episodes of violence, 4.6% by building collapses, 3.4% by bomb explosions and the remaining 10.8% by all other causes.

Table 1 presents the percentages of casualties for each cause of major incident according to the pre-hospital outcome.

**Table 1:** Percentages of casualties for each cause of major incident according to the pre-hospital outcome

	RTC	Fire	Building collapse	Violence	Bomb explosion	Other causes
Dead %	6.7	6.9	12.9	28.9	11.1	23.3
First aid %	28.9	9.8	24.2	10.7	4.6	23.3
Hospital %	64.4	83.3	62.9	60.4	84.3	53.4

There was no difference between the prevalences of daytime and night-time road traffic crashes during the 6-month study period.

Average first responder time from call to arrival at the scene was 7 [5-12] min (median [IQR]). Daytime median response time was 7 [5-12] min, while night-time median response time was 8 [5-12] min. There was no significant difference between day and night response times (*p *= 0.62).

During the 438 emergency calls a total of 1,590 transportation resources were used. Of these, 52.8% were emergency service ambulances, which are BLS Basic Life Support only, 14.7% were rescue vehicles, 26.6% were fire department trucks and 4.1% were water bowsers, while the remaining 1.8% were other means of transportation. During RTC interventions 82.4% of resources were ambulances, 14.9% were rescue vehicles and 2.7% were fire trucks. During fires, intervention ambulances represented 21.6%, rescue vehicles 11.6%, fire trucks 56.3% and water bowsers 10.5% of the resources used.

## Discussion

Half of all major incidents and two thirds of all deaths in major incidents are due to road traffic crashes. Interestingly, most RTCs were grouped in the high population density rural districts. Major RTCs were less common in the industrialized districts, whereas fires were the most frequent major incident in the major cities. However, the number of casualties due to fire incidents was significantly less than that of RTCs. After RTCs the second leading cause of mortality was violence (7.7%), which was specifically interpersonal violence including gunshots. Interpersonal violence in rural Punjab can include the use of firearms or the more traditional method of beating victims with wooden sticks or iron rods. This method leads to severe blunt trauma and head injuries, and in some cases is more lethal than firearm injuries. Mass lynching is also not unheard of in Punjab and was part of this 7.7% of major incidents.

Some other causes of major incidents appeared that may be particular to a developing country like Pakistan where buildings collapse (6.5%). This category included both well and wall collapses. Well collapses are indigenous to Southern Punjab where while digging wells for water the whole structure may suddenly collapse because of soft sand, and the men digging them may find themselves buried under tons of mud. Gas leakages and gas cylinder explosions resulted in 2.6% of major incidents. These explosions are often due to faulty pipes or old, non-calibrated gas cylinders. Drowning (3%) was also concentrated in three main districts; in fact 50% were only in the Sialkot district at one specific site. All drowning victims were young males on tourist excursions to dams and canals. With the recent increase in terrorism across Pakistan, bomb explosions were another cause of major incidents.

The high mortality associated with road traffic crashes has previously been demonstrated by other authors and other studies in developing countries. National Health Survey Pakistan (1994) has already established the high burden of injuries caused by RTCs in Pakistan [16-20]. In Pakistan, legal rules for traffic are laid down in three basic documents:

• Motor Vehicle Ordinance 1965

• West Pakistan Motor Vehicle Rules 1969

• Pakistan Highway Code

The driving codes include point penalties for speeding, driving while intoxicated, not using seat belts and helmets, and in some cases mobile phone use as well [21]. However, implementation and proper enforcement of these laws remain problematic [22].

A Fire and Safety Law is currently being drafted by policy makers that covers prevention and mitigation measures, including marking fire exits in buildings and having fire extinguishers. However, the rest of the major incident causes mentioned in this study have not received any official recognition or governmental support to date. Keeping the findings of this study in view, the value of injury prevention cannot be overemphasized in a low-income country such as Pakistan.

The high death rate of 10.4% on the scene -- that is, the mortality even before the arrival of the EMS -- implies that prevention rather than cure should be the aim. What is concerning about most of these incidents is that they were potentially preventable. In the developed countries, mechanisms of injury have been well studied, including road traffic injuries, injuries in the home, drowning, fires, violence and suicide; however, the same cannot be said for the developing world. The solution does not however lie completely in importing injury control techniques from developed to developing countries as they will not accomplish much because of the differing causes of injury and the different social and economic contexts in which they occur. Instead, there is a need for local adaptations and even the development of innovative strategies for which of course more directed research is needed [23].

The authors understand that there are certain limitations of the paper.

1. Selection bias - possibly some of the incidents were not handled by the rescue service.

2. Reporting bias - Incidents on the major roads are more likely to be reported. The victims of other incidents in urban areas are often picked up early by taxis or bystanders, and thus the need for calling 1122 is less.

3. This is not a study from the entire province as the data were only collected from 24 out of 35 districts. At the time of this study, only 24 districts had the proper guidelines to report major incidents.

## Conclusion

RTCs are the most common reason for major incident responses in developing countries such as Pakistan. Fire and violence are the second and third leading causes of major incidents. RTCs cause the most injuries, followed by violence. Policies for prevention of these injuries are critical as 10% of the people die even before the EMS gets there. This study is of benefit to policy makers and public health professionals for injury prevention policy making and will hopefully be a stimulus for initiating injury prevention programs and initiatives in the country.

## Competing interests

The fourth author of this paper is Director General of Rescue 1122, Punjab Emergency Service. No competing interests were identified for other authors.

## Authors' contributions

HW, LC, JR, RS conceived the study design. RN and HW conducted data collection. HW, JR, and LC conducted analyses. All authors participated in interpretation of results. HW wrote the first manuscript. LC, JR, and RN critically reviewed the manuscript. All authors read and approved the final manuscript
